# Double-Stranded RNA-Dependent Protein Kinase Regulates the Motility of Breast Cancer Cells

**DOI:** 10.1371/journal.pone.0047721

**Published:** 2012-10-24

**Authors:** Mei Xu, Gang Chen, Siying Wang, Mingjun Liao, Jacqueline A. Frank, Kimberly A. Bower, Zhuo Zhang, Xianglin Shi, Jia Luo

**Affiliations:** 1 Department of Internal Medicine, University of Kentucky College of Medicine, Lexington, Kentucky, United States of America; 2 Pathophysiological Department, School of Basic Medicine, Anhui Medical University, Hefei, Anhui, China; 3 Graduate Center for Toxicology, University of Kentucky College of Medicine, Lexington, Kentucky, United States of America; University of Sheffield, United Kingdom

## Abstract

Double-stranded RNA (dsRNA)-dependent protein kinase (PKR) is an interferon-induced protein kinase that plays a central role in the anti-viral process. Due to its pro-apoptotic and anti-proliferative action, there is an increased interest in PKR modulation as an anti-tumor strategy. PKR is overexpressed in breast cancer cells; however, the role of PKR in breast cancer cells is unclear. The expression/activity of PKR appears inversely related to the aggressiveness of breast cancer cells. The current study investigated the role of PKR in the motility/migration of breast cancer cells. The activation of PKR by a synthesized dsRNA (PIC) significantly decreased the motility of several breast cancer cell lines (BT474, MDA-MB231 and SKBR3). PIC inhibited cell migration and blocked cell membrane ruffling without affecting cell viability. PIC also induced the reorganization of the actin cytoskeleton and impaired the formation of lamellipodia. These effects of PIC were reversed by the pretreatment of a selective PKR inhibitor. PIC also activated p38 mitogen-activated protein kinase (MAPK) and its downstream MAPK-activated protein kinase 2 (MK2). PIC-induced activation of p38 MAPK and MK2 was attenuated by the PKR inhibitor and the PKR siRNA, but a selective p38 MAPK inhibitor (SB203580) or other MAPK inhibitors did not affect PKR activity, indicating that PKR is upstream of p38 MAPK/MK2. Cofilin is an actin severing protein and regulates membrane ruffling, lamellipodia formation and cell migration. PIC inhibited cofilin activity by enhancing its phosphorylation at Ser3. PIC activated LIM kinase 1 (LIMK1), an upstream kinase of cofilin in a p38 MAPK-dependent manner. We concluded that the activation of PKR suppressed cell motility by regulating the p38 MAPK/MK2/LIMK/cofilin pathway.

## Introduction

Double-stranded RNA (dsRNA)-dependent protein kinase (PKR) is a 551 amino acid serine/threonine protein kinase and is ubiquitously expressed in mammalian cells. PKR was initially identified as an essential element in interferon-induced anti-viral processes. PKR is activated by viral dsRNA intermediates during infection via a mechanism involving autophosphorylation. Once activated, the enzyme phosphorylates the α-subunit of protein synthesis initiation factor eIF2, thereby inhibiting translation [1]. PKR also mediates the activation of signal transduction pathways by proinflammatory stimuli, including bacterial lipopolysaccharide (LPS), tumor necrosis factor-α (TNF-α) and interleukin 1 (IL-1) [1]. PKR can be directly activated by its cellular activator PACT [2]. In addition, serum deprivation, disruption of intracellular Ca^2+^ homeostasis, oxidative stress and endoplasmic reticulum (ER) stress also stimulate PKR activity [3], [4]. Active PKR regulates diverse downstream substrates and signaling pathways, such as NFκB, p53, protein phosphatase 2A (PP2A), MAPK and STAT1/STAT3 signaling [3]. PKR has been implicated in the regulation of cell proliferation, apoptosis, differentiation and transformation [5], [6]. In general, activation of PKR results in the inhibition of cell proliferation or the induction of apoptosis and translational suppression; therefore, PKR is considered a “tumor suppressor” and considerable attention has been paid to the PKR pathway for its anti-tumor potential [3], [7].

In contrast to its potential role as a tumor suppressor, PKR is over-expressed in a number of human cancers including breast cancers [8]–[11]. A higher level of PKR is observed in human invasive ductal breast carcinomas than surrounding normal mammary tissues [9]. In addition, much more PKR is expressed in mammary carcinoma cell lines compared to non-transformed mammary epithelial cell lines [4], [11]. However, it appears the expression levels of PKR are inconsistent with its activity detected in mammary tumor cells and epithelial cells. The role of PKR in breast cancer cells is unclear. Among human breast cancer cell lines, it appears that the aggressiveness is inversely related to PKR expression/activity [8]. For example, MCF-7, a minimally invasive breast cancer cell line expresses high levels/activity of PKR, while MDA-MB231, a highly invasive breast cancer cell line, has low levels/activity of PKR [8]. We therefore hypothesized that PKR plays a role in the aggressiveness in breast cancer cells.

Tumor cell motility/migration is the hallmark of invasion and an essential step in metastasis. Cell motility/migration is a complex biological process that is regulated by a myriad of molecular/cellular events including cytoskeleton reorganization, membrane ruffling and lamellipodia/filopodia formation [12], [13]. In this study, we investigated the involvement of PKR in regulation of cell motility/migration. We demonstrated that the activation of PKR inhibited the motility/migration of breast cancer cells. We further showed that activation of PKR suppressed cofilin activity. Cofilin is an F-actin severing protein that regulates actin depolymerization, a process required for the changes in cell shape and the formation of lamellipodia during migration [14], [15]. Cofilin plays an important role in the invasion and metastasis of breast cancer cells [16], [17]. We further delineated a signal transduction pathway that mediated PKR regulation of cofilin activity.

## Materials and Methods

### Materials

Human plasma fibronectin was obtained from Chemicon International (Temecula, CA). Anti-phospho-eIF2α (Ser51) antibody and Lipofectamine 2000 were purchased from Invitrogen Corporation (Carlsbad, CA). Antibodies for phospho-p38 MAPK, phospho-cofilin, cofilin, phospho-LIMK, LIMK, phospho-MAPKAPK2 (MK2) and MK2 were purchased from Cell Signaling Technology Inc. (Beverly, MA). Antibodies for PKR, GAPDH, α-actin, eIF2α and p38α were purchased from Santa Cruz Biotechnology (San Cruz, CA). Anti-phospho-PKR antibody was obtained from Abcam (Cambridge, MA). Phalloidin 488, Alex Fluor-labeled secondary antibodies and Prolong Gold anti-fade reagent were obtained from Invitrogen Molecular Probes (Eugene, OR). MTT assay kit was purchased from Roche Molecular Biochemicals (Indianapolis, IN). Transwells were obtained from Costar Corp. (Acton, MA). Polyriboinosinic acid [poly I]-polyribocytidylic acid [poly C] (PIC) was purchased from InvivoGen (San Diego, Ca). Inhibitors for PKR, p38 MAPK, ERK and JNK were obtained from Millipore (Billerica, MA). All other chemicals were obtained from Sigma-Aldrich (St. Louis, MO).

**Figure 1:**
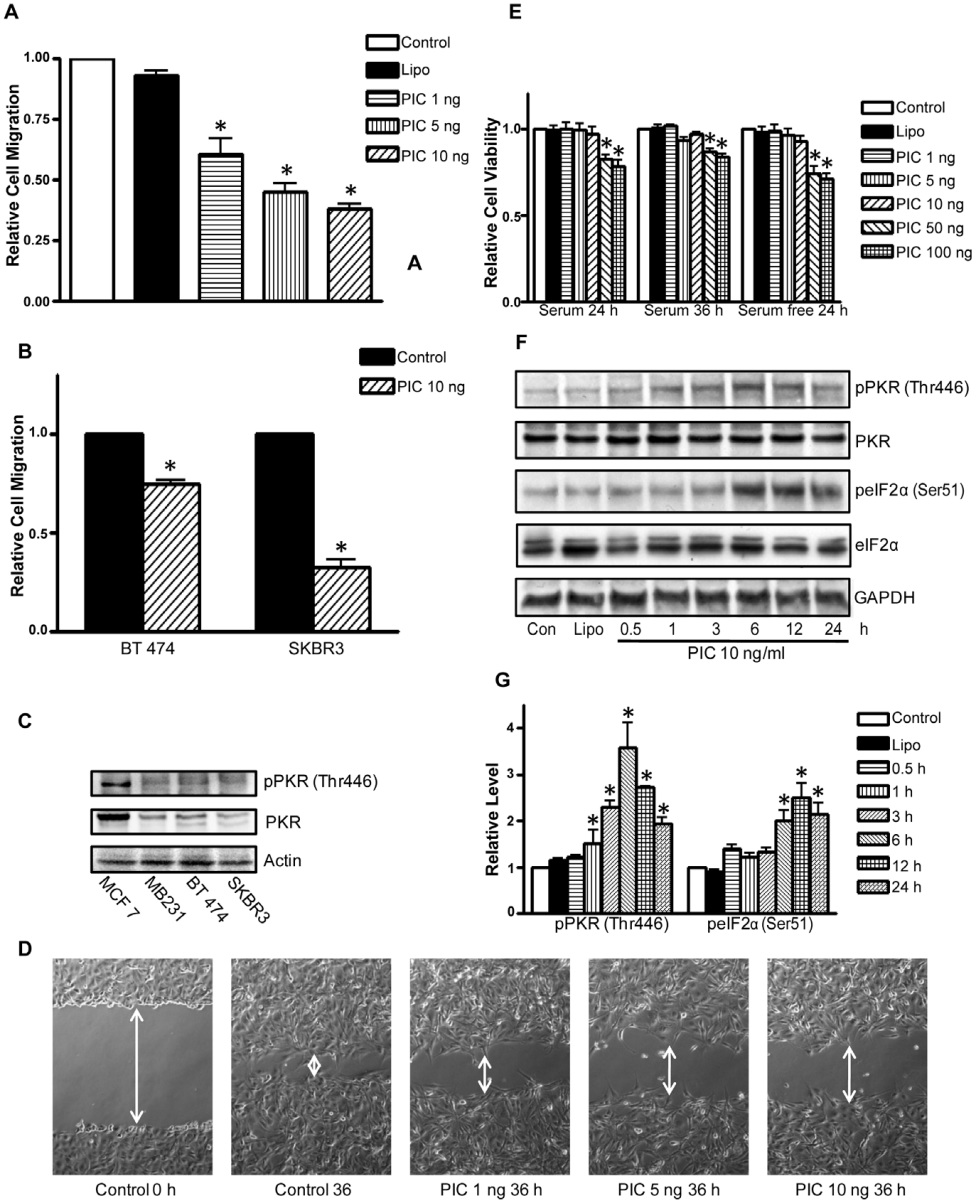
Activation of PKR inhibits the motility of breast cancer cells. **A**: MDA-MB231 cells (5x10^4^) were placed into the upper compartments of migration chambers (transwells) in the presence of PIC (0, 5 and 10 ng/ml). The transwells were incubated at 37°C in 5% CO_2_ overnight. The number of migrated MDA-MB231 cells was measured as described under the Materials and Methods. **B**: The number of migrated BT474 and SKBR3 cells in the presence of PIC treatment (0 or 10 ng/ml) was determined as described above. **C**: The expression of phosphorylated PKR and total PKR in MCF7, MB231, BT474 and SKRB3 cells were determined by immunoblotting. **D**: MDA-MB231 cells were exposed to PIC (0, 5 and 10 ng/ml) for 36 h and cell migration was determined by wound healing migration assay as described under the Materials and Methods. **E**: MDA-MB231 cells were exposed to PIC (0, 5, 10, 50 or 100 ng/ml) with/without serum for 24 and 36 h. The cell viability was determined with MTT assay. The number of viable cells after PIC treatment was presented relative to untreated controls. **F**: MDA-MB231 cells were treated with PIC (0 or 10 ng/ml) for indicated time courses. Cell lysates were collected for immunoblotting analysis of the phosphorylation/expression of PKR and eIF2α. The expression of GAPDH served as a loading control. **G**: The relative levels of pPKR and pelF2α were quantified as described under the Materials and Methods and normalized to the expression of PKR and elF2α, respectively. Each datum point was the mean ± SEM of three independent experiments. * denotes a statistically significant difference from untreated controls (p<0.05).

### Cell Culture and PIC Treatments

MDA-MB231, BT474 and SKBR3 human breast cancer cell lines were obtained from American Type Culture Collection (ATCC, Manassas, VA). MDA-MB231 breast cancer cells were grown in DMEM medium containing 10% fetal bovine serum (FBS) and 1% antibiotics-antimycotic (Gibco, Grand island, NY) at 37°C with 5% CO_2_. MDA-MB231 cells were used for most of the experiments because they have high invasive potential and low PKR expression/activity. BT474 cells were grown in RPMI 1640 medium containing 10% FBS, 1% antibiotics-antimycotic and 10 µg/ml insulin at 37°C with 5% CO_2_. SKBR3 cells were grown in IMEM medium containing 10% FBS and 1% antibiotics-antimycotic at 37°C with 5% CO_2_. PIC, a synthetic analog of dsRNA, was used to activate PKR. For PIC treatment, Lipofectamine 2000 was included to facilitate PIC entry into the cells. For controls, cells were treated with Lipofectamine 2000 alone. The concentrations of PIC for this study ranged from 1–100 ng/ml. For treatment with PKR inhibitor, cells were pretreated with the PKR inhibitor (500 nM) for 24 hours prior to PIC exposure. For treatment with p38 MAPK inhibitor (SB203580), cells were pretreated with SB203580 (5 µM) for 2 hours, then exposed to PIC.

**Figure 2:**
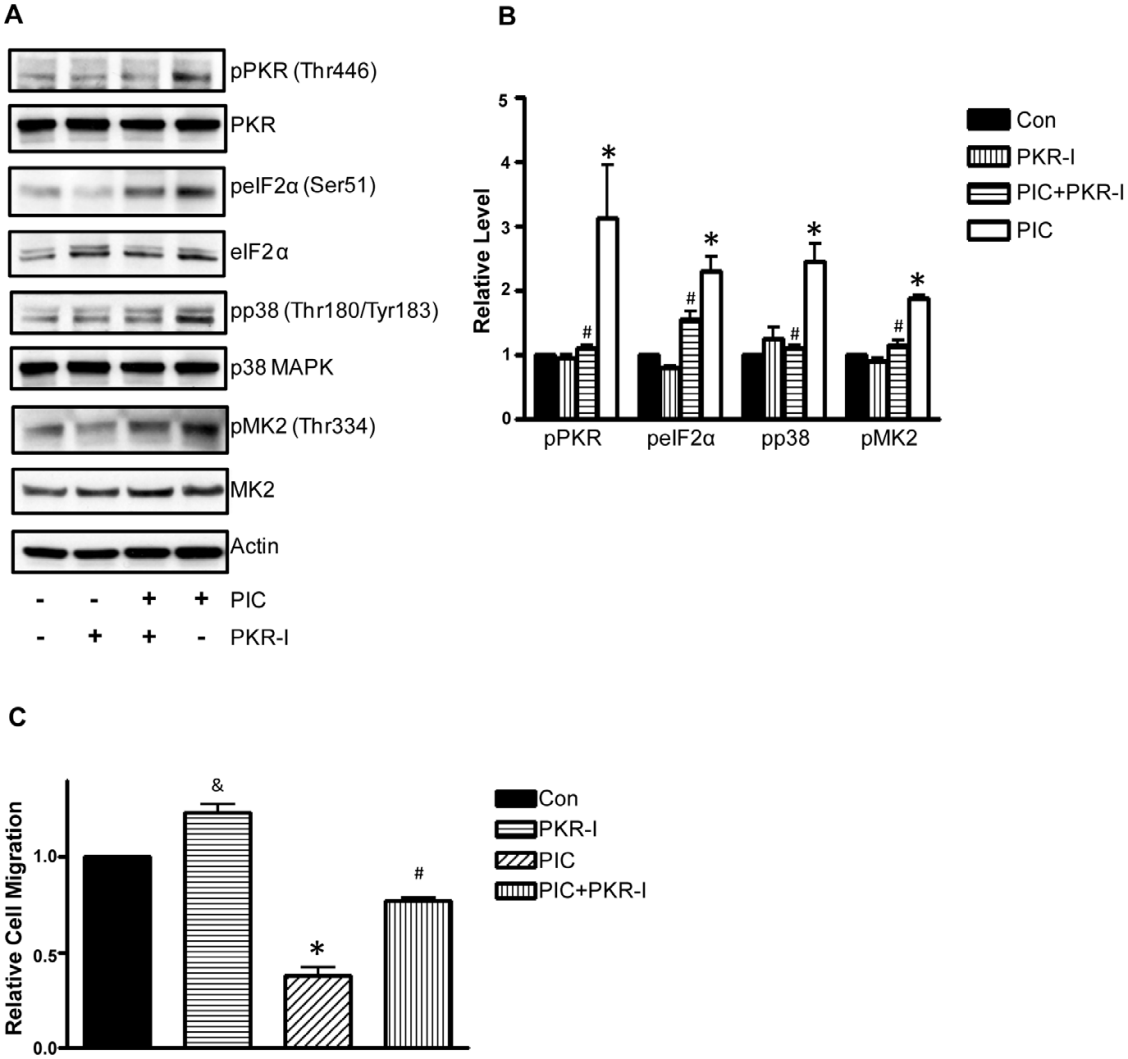
Effect of PKR inhibitor on PIC-induced inhibition of cell migation. **A**: MDA-MB231 cells were pretreated with a selective PKR inhibitor (PKR-I, 500 nM) for 24 hours followed by PIC (0 or 10 ng/ml) exposure for 6 hours. Cell lysates were collected for immunoblotting analysis of the phosphorylation/expression of PKR, eIF2α, p38 MAPK and MK2. The expression of actin served as a loading control. **B**: The relative levels of pPKR, pelF2α, pp38 and pMK2 were quantified as described under the Materials and Methods and normalized to the expression of PKR, elF2α, p38 MAPK and MK2, respectively. * denotes a statistically significant difference from untreated groups. # denotes a significant difference from PIC-treated groups. **C**: MDA-MB231 cells were pretreated with PKR-I (500 nM) for 24 h then placed into the upper compartments of migration chambers in the presence of PIC (0 or 10 ng/ml). The number of MDA-MB231 cells that migrated through the transwells was measured as described under the Materials and Methods. The experiment was replicated three times. * denotes a statistically significant difference from non-PIC-treated groups. # denotes a significant difference from PIC-treated groups. & denotes a significant difference from the untreated group.

### PKR siRNA Transfection

Transient transfection of PKR siRNA (San Cruz, CA) was performed with Lipofectamine 2000 according the manufacturer’s protocol. Briefly, MB231 cells were treated by control siRNA (con siRNA) or PKR siRNA at a concentration of 200 nM in the presence of Lipofectamine 2000. After 48 hours of incubation, cells were exposed to PIC and cell signaling events or cell migration were assayed.

**Figure 3:**
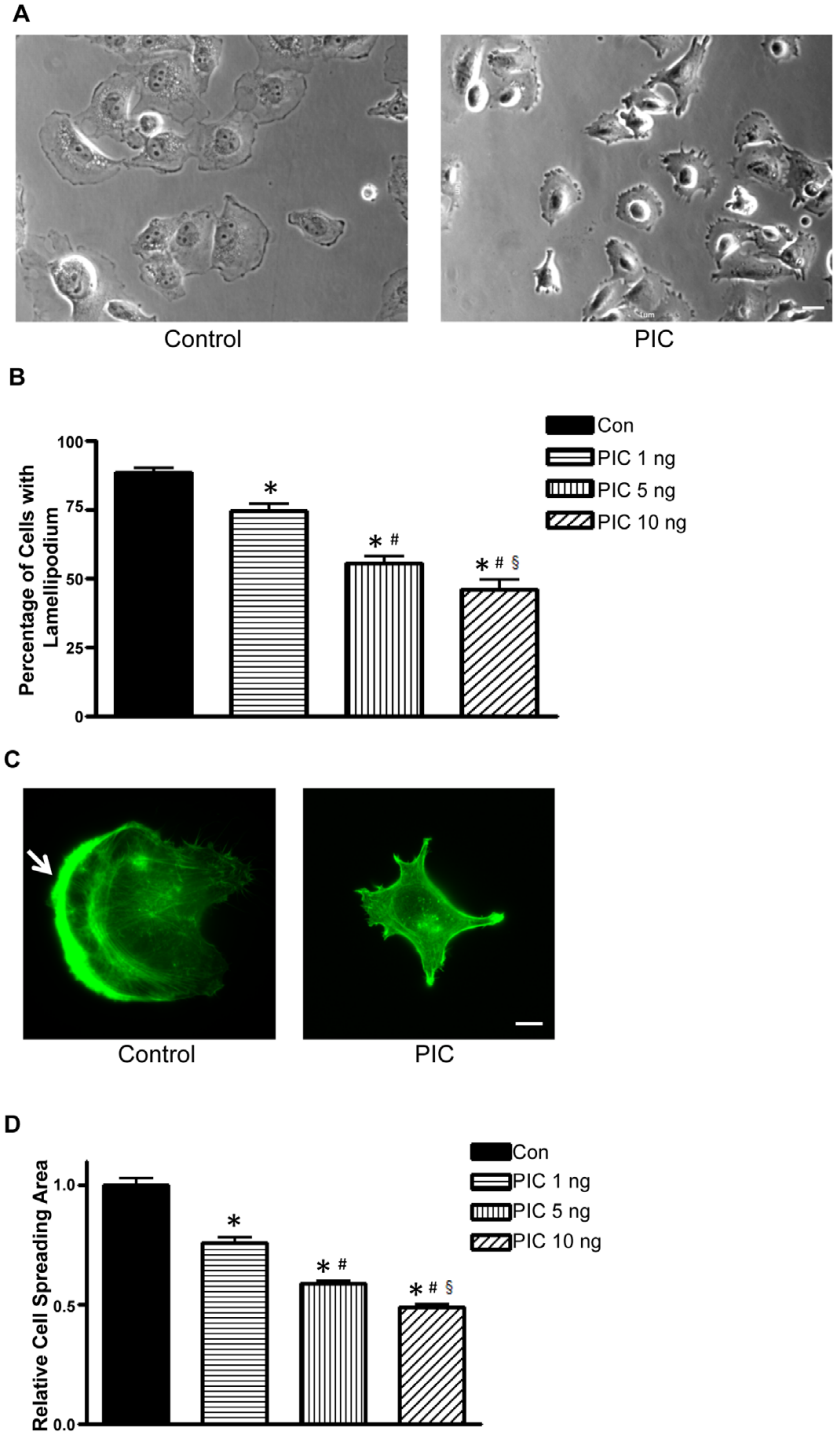
Activation of PKR impairs lamellipodia formation. MDA-MB231 cells were treated with PIC (0, 1, 5 or 10 ng/ml) for 12 hours, and then equal amounts of cells were seeded on fibronectin-coated culture wells, allowing attachment for 3 hours. **A**: After attaching, the phase-contrast images were captured using a Zeiss Axiovert 40C photomicroscope. The images of cells treated with PIC (0 or 10 ng/ml) are presented. Scale bar  = 50 µm. **B**: Cells with extended leading areas (lamellipodia) were counted in ten randomly selected fields in each treatment group. The percentage of cells with lamellipodia was determined. **C**: MDA-MB231 cells were treated with PIC (0 or 10 ng/ml) for 12 hours. The distribution of the actin cytoskeleton was detected by fluorescent staining (Alexa Fluor 488 Phalloidin) as described under the Materials and Methods. The arrow indicates the lamellipodia (the leading edge). Scale bar  = 10 µm. **D**: MDA-MB231 cells were treated with PIC (0, 1, 5 or 10 ng/ml) for 12 hours, and then equal amounts of cells were seeded on fibronectin-coated culture wells, allowing attachment for 3 hours. Cell spreading areas were measured randomly for at least 25 cells for each treatment group. The experiment was replicated three times. Each datum point was the mean ± SEM of three independent experiments. * denotes a significant difference from untreated controls. # denotes a significant difference from PIC (1 ng/ml)-treated groups. § denotes a significant difference from PIC (5 ng/ml)-treated groups (p<0.05).

### Cell Migration

Cell migration was analyzed using a Transwell Migration System (Costar) as described before [18]. Briefly, cells (5×10^4^ cells) were placed into the upper chambers (Transwells with 8.0 µm pore size) in serum free medium in the presence or absence of PIC. Culture medium containing 10% FBS was added into the lower compartment of the chambers and served as a chemoattractant for the cells. The chambers were cultured at 37°C in 5% CO_2_ overnight. The migrated cells were fixed in 3.7% paraformaldehyde and stained with 0.5% crystal violet in 2% ethanol. Membranes were washed and the dye was eluted with 10% acetic acid. Absorbance was measured at 595 nm using a microtiter platereader (Beckman coulter). PIC or chemical inhibitors were only added to the upper chambers.

**Figure 4:**
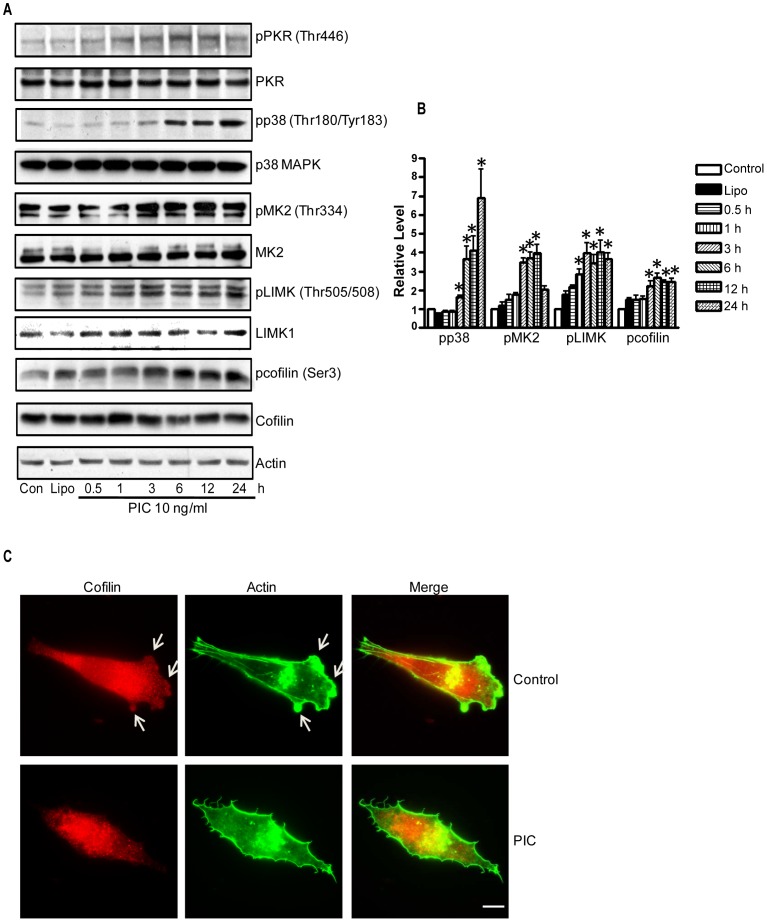
Activation of PKR inhibits cofilin. **A**: MDA-MB231 cells were treated with PIC (0 or 10 ng/ml) for indicated times. There were two controls; cells received no treatment (con) or cells were treated with Lipofectamine 2000 (Lipo). Cell lysates were collected for immunoblotting analysis of the phosphorylation/expression of PKR, p38 MAPK, MK2, LIMK1 and cofilin. The expression of actin served as a loading control. **B**: The relative levels of pp38, pMK2, pLIMK and pcofilin were quantified as described under the Materials and Methods and normalized to the expression of p38 MAPK, MK2, LIMK1 and cofilin respectively. * denotes a significant difference from untreated controls. **C**: MDA-MB231 cells were exposed to PIC (0 or 10 ng/ml) for 6 hours, and then cells were seeded on fibronectin-coated culture wells, allowing attachment for 3 hours. The expression of cofilin and actin was detected by immunofluorescent staining as described under the Materials and Methods. Arrows indicate the lamellipodia. Scale bar  = 10 µm. These experiments were replicated three times.

### Wound Healing Migration Assay

The wound healing migration assay was performed as described previously [18]. MDA-MB231 cells were grown on 35 mm dishes to 100% confluence and then scratched to form a wound using sterile pipette tips. The cells were then treated with PIC (0, 1, 5 or 10 ng/ml) for 36 hours. The images were recorded using a Zeiss Axiovert 40C photomicroscope.

**Figure 5:**
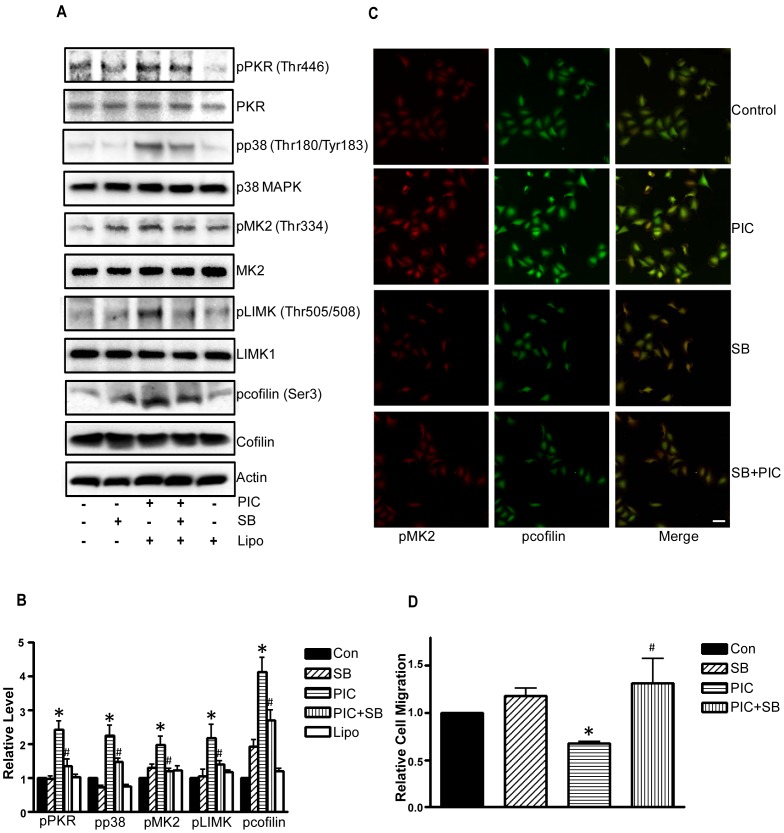
Effect of p38 MAPK inhibitor on PIC-stimulated cell signaling. MDA-MB231 cells were pretreated with a selective p38 MAPK inhibitor (SB203580, 5 µM) for 2 hours followed by PIC (0 or 10 ng/ml) treatments for an additional 6 hours. **A**: After the treatment, cell lysates were collected for immunoblotting analysis of the phosphorylation/expression of PKR, p38 MAPK, MK2, LIMK1 and cofilin. The expression of actin served as a loading control. **B**: The relative levels of pPKR, pp38, pMK2, pLIMK and pcofilin were quantified as described under the Materials and Methods and normalized to the expression of PKR, p38 MAPK, MK2, LIMK1 and cofilin respectively. * denotes a significant difference from untreated controls. **C**: The expression of phospho-cofilin and phospho-MK2 was visualized by immunofluorescent staining as described under the Materials and Methods. Scale bar  = 100 µm. **D**: MDA-MB231 cells were pretreated with SB203580 (5 µM) for 2 hours then placed into the upper compartments of migration chambers (transwells) in the presence of PIC (0 or 10 ng/ml). The number of MDA-MB231 cells that migrated through the transwells was determined as described under the Materials and Methods. The experiment was replicated three times. * denotes a statistically significant difference from controls. # denotes a significant difference from PIC-treated groups.

**Figure 6:**
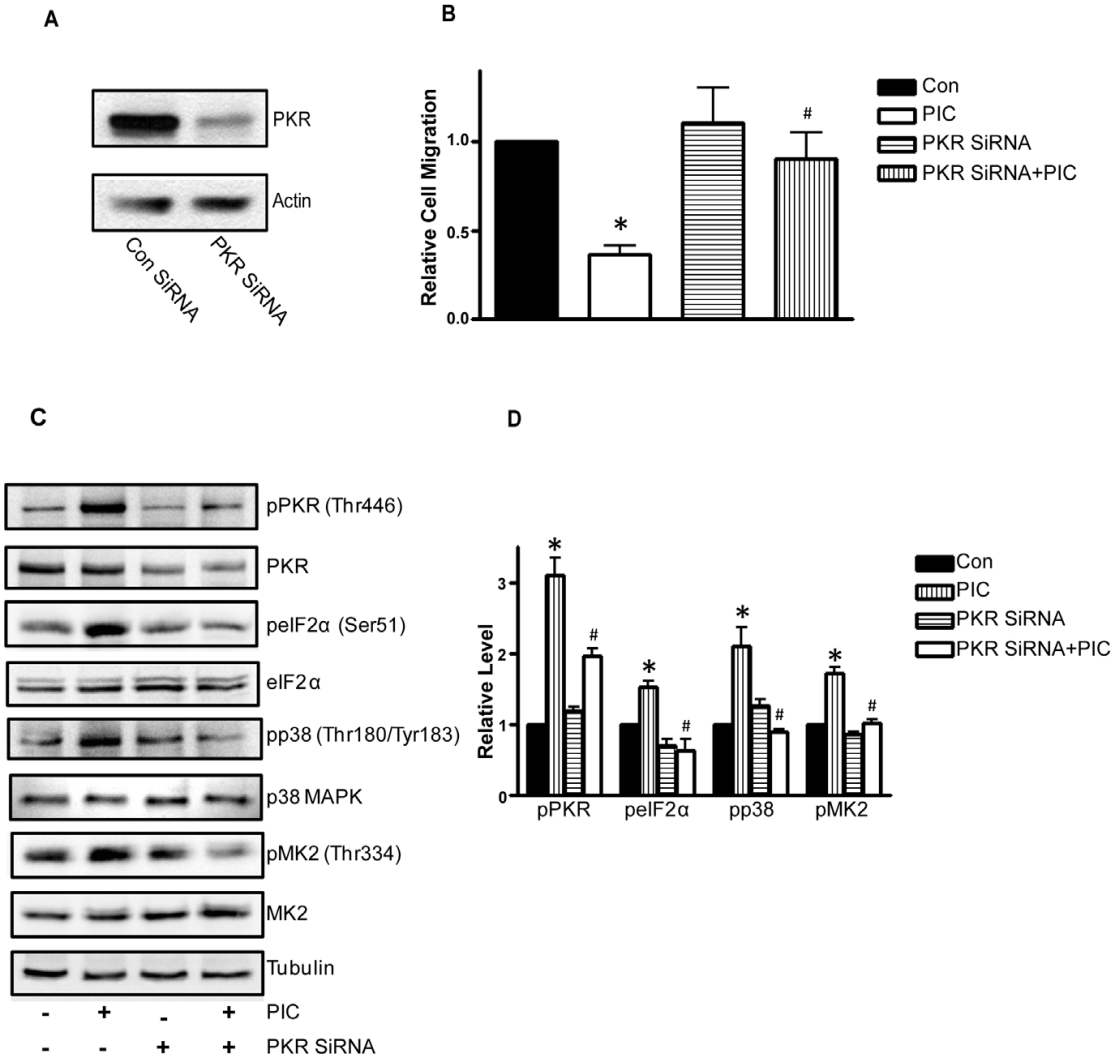
Effect of PKR siRNA on PIC-regulated cell migation. MDA-MB231 cells were transfected with control siRNA or PKR siRNA for 48 h. **A**: Following transfection, cell lysates were collected and the expression PKR was determined by immunoblotting. **B**: Following transfection, MDA-MB231 cells were placed into the upper compartments of the migration chambers in the presence of PIC (0 or 10 ng/ml). The number of MDA-MB231 cells that migrated through the transwells was measured as described under the Materials and Methods. * denotes a statistically significant difference from controls. # denotes a significant difference from PIC-treated groups. **C**: Following transfection, MDA-MB231 cells were exposed to PIC (0 or 10 ng/ml) for 6 hours. Cell lysates were collected for immunoblotting analysis of the phosphorylation/expression of PKR, eIF2α, p38 MAPK and MK2. The expression of tubulin served as a loading control. **D**: The relative levels of pPKR, pelF2α, pp38 and pMK2 were quantified as described under the Materials and Methods and normalized to the expression of PKR, elF2α, p38 MAPK and MK2, respectively. The experiment was replicated three times. * denotes a statistically significant difference from controls. # denotes a significant difference from PIC-treated groups.

### MTT Assay

The MTT assay was employed to determine the number of viable cells in culture. Briefly, the cells were plated into 96-well plates and exposed to PIC for 24 hours. After the treatment, 10 µl of MTT reagent was added into each well and the plates were incubated at 37°C for 4 hours. The cultures were solubilized and spectrophotometric absorbance was measured at 595 nm using a microtiter platereader.

**Figure 7:**
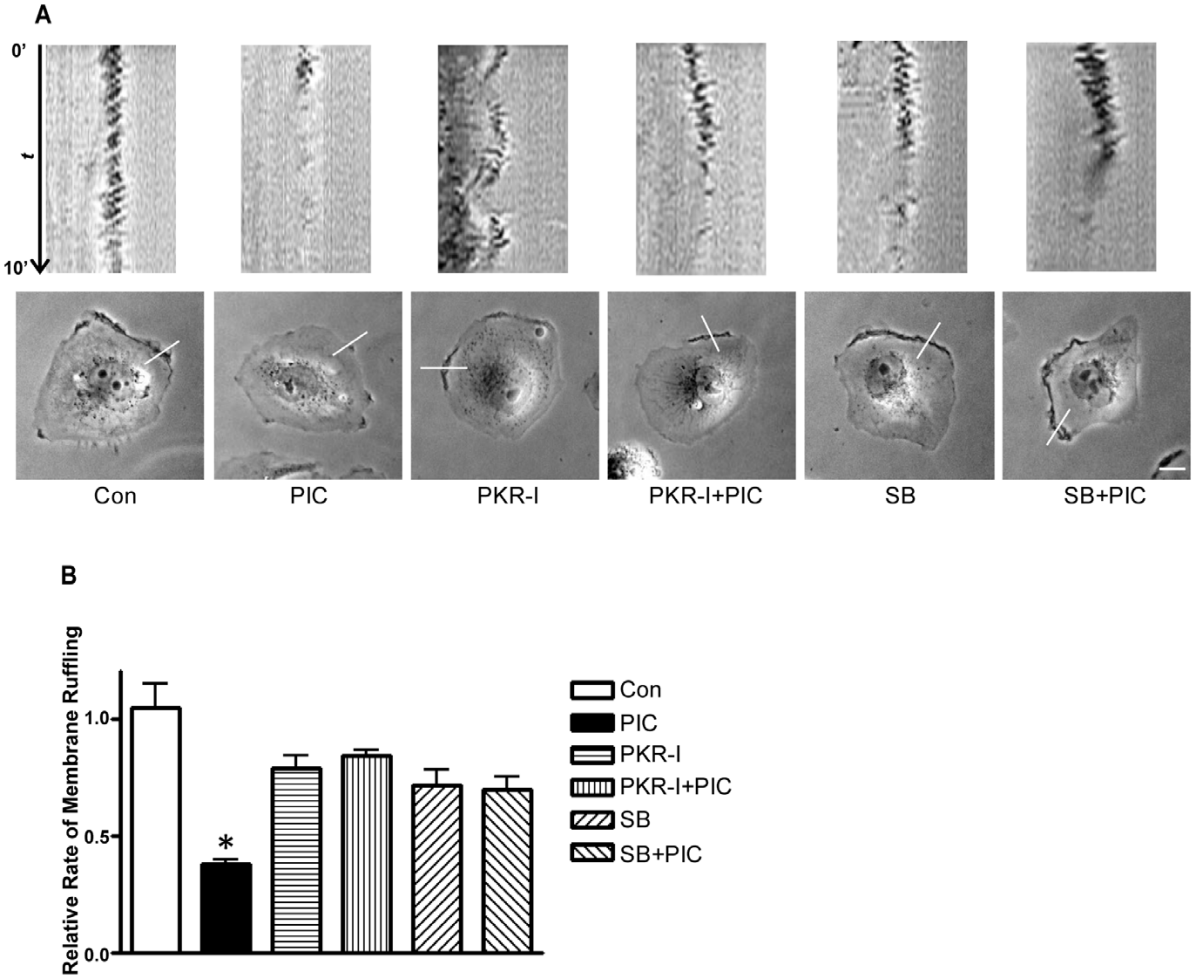
Activation of PKR inhibits dynamic cell membrane ruffling. **A**: MDA-MB231 cells were pretreated with DMSO (control), PKR-I (500 nM) or SB203580 (5 µM) for indicated hours, then followed by the treatment of PIC (10 ng/ml) for 6 hours. Top panel: Phase-contrast frames were acquired every 10 seconds for 10 minutes on a time-lapse microscope using a 60X oil-immersion lens. Scale bar  = 10 µm. Bottom panel: The corresponding kymographs of the movie generated along a line transecting the cell membrane on the lamellipodia are presented. **B**: Relative rate of cell membrane ruffling was calculated from kymographs. The experiments were replicated five times. * denotes a statistically significant difference from all other groups (p<0.05).

### Analysis of Cell Morphology

Cell adhesion to fibronectin was analyzed as described previously [19], [20]. Briefly, cells were exposed to PIC for 12 hours. After exposure, cells (5×10^4^/well) were seeded on fibronectin (10 µg/ml)-precoated plates, allowing attachment for 3 hours at 37°C with 5% CO_2_. Non-adherent cells were removed by washing with PBS. The attached cells were fixed with 3.7% paraformaldehyde for 10 min, then washed 3 times in PBS. The phase-contrast images were captured using a Zeiss Axiovert 40C photomicroscope. Cells with protruded leading areas (lamellipodia) were counted in ten randomly selected fields in each treatment group. Cell spreading areas were measured using Motic Images Plus 2.0 ML software (Motic, Canada).

**Figure 8:**
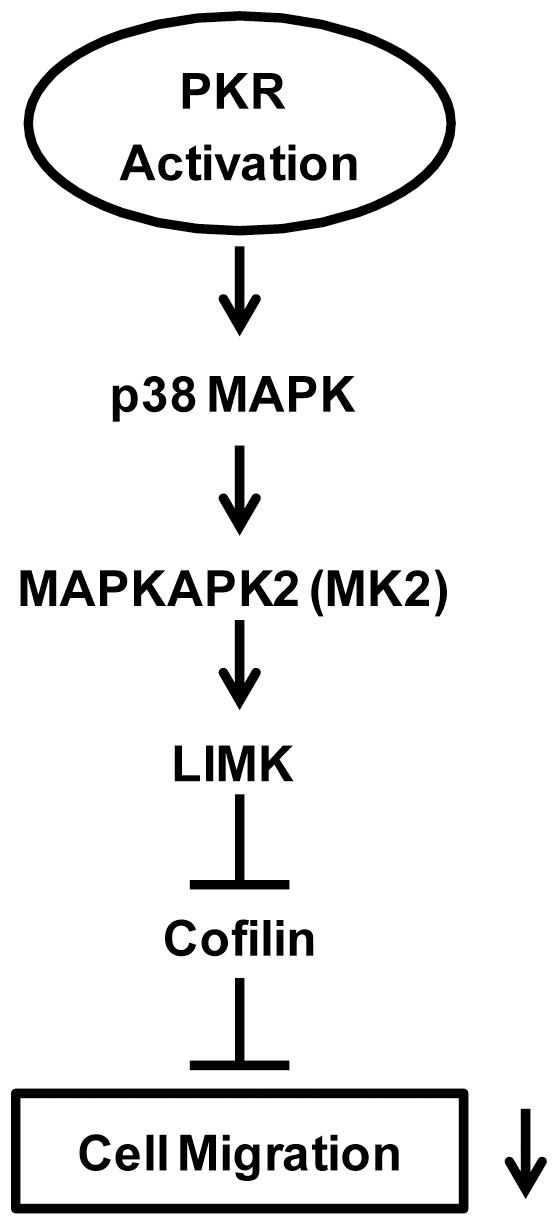
Schematic illustration of PKR-mediated cell signaling that regulates cell migration. The activation or high expression of PKR results in the activation of p38 MAPK/MK2 which stimulates the phosphorylation of LIMK. The activated LIMK inhibits cofilin, resulting in the suppression of cell migration.

### Immunofluorescence Microscopy

The procedure for immunofluorescence microscopy has been previously described [21]. Briefly, after treatments, cells were fixed with 3.7% paraformaldehyde for 10 min, washed 3 times in PBS and permeabilized with 0.5% Triton X-100 for 5 min. Cells were blocked with 5% BSA and incubated with primary antibodies for 1 hour. The concentrations of primary antibodies were: anti-cofilin, 1∶100; anti-phospho-cofilin, 1∶100; anti-phospho-MAPKAPK2, 1∶100; and phalloidin, 1∶200. After the incubation, cells were washed and treated with Alexa Fluor-labeled secondary antibodies and rinsed several times with PBS. Coverslips were mounted with Prolong Gold anti-fade reagent and immunofluorescence images were examined with an Olympus 1X81 inverted fluorescent microscope with the same exposure time, detector gain and amplifier offset.

### Immunoblotting

The procedure for immunoblotting has been previously described [19]. Briefly, after PIC treatments, cells were lysed in modified RIPA buffer (150 mM NaCl, 50 mM Tris, 1% NP-40, 0.25% sodium deoxycholate, 1 mM sodium vanadate, 1 mM phenylmethanesulfonyl fluoride (PMSF), 5 µg/ml of aprotinin, and 2 µg/ml of leupeptin). Samples were separated by centrifugation at 10,000 rpm for 10 min at 4°C. Proteins were resolved in SDS-PAGE and the separated proteins were transferred to nitrocellulose membranes. The membranes were probed with indicated primary antibodies, followed by the appropriate secondary antibodies and developed by enhanced chemiluminescence. The images of immunoblots were documented using Gel Logic 2200 Pro (Carestream Health, Rochester, NY). The intensity of specific proteins was quantified using Carestream Molecular Image Software.

### Time-lapse Microscopy

The rate of cell membrane ruffling was determined by time-lapse microscopy. After treated with PIC for 12 hours in the presence or absence of inhibitors, cells were trypsinized and seeded on fibronectin precoated glass-bottom dishes in medium containing 20 mM HEPES. Cells were allowed to attach for 3 hours at 37°C with 5% CO_2_. Cells were maintained at 37°C and recorded by a phase-contrast time-lapse video program using an Olympus 1X81 inverted fluorescent microscope with a 60X oil immersion lens. The recording was performed at 10-second intervals for 10 min. Kymographs were generated from time-lapse video images using ImageJ software (NIH) as previously described [14]. Five or eight 1-pixel-thick lines were drawn across the cell leading edges and the pixel intensities along each line were combined to create the kymographs. To determine the dynamic frequency of cell membrane ruffling, the perceived changes of waves in 10 min on kymographs were counted manually and presented relative to the control groups.

### Statistics

Differences among treatment groups were analyzed using analysis of variance (ANOVA). Differences in which *p* was less than 0.05 were considered statistically significant. In cases where significant differences were detected, specific *post-hoc* comparisons between treatment groups were examined with Student-Newman-Keuls tests.

## Results

### Activation of PKR Inhibits the Motility of Breast Cancer Cells

We first examined the effect of PIC on the motility/migration of breast cancer cells. As shown in Fig. 1A, PIC suppressed the migration of MDA-MB231 cells which was determined by transwell assay in a concentration-dependent manner. Even at 1 ng/ml, PIC significantly inhibited the migration of MDA-MB231 cells. Similar effects of PIC were observed in other aggressive breast cancer cells (BT474 and SKBR3) (Fig. 1B). We examined the expression/phosphorylation of PKR in different breast cancer cell lines. As shown in Fig. 1C, the levels of PKR expression/phosphorylation were much lower in more aggressive breast cancer cell lines (MDA-MB231, BT474 and SKBR3) than in the less aggressive line (MCF-7). PIC-mediated inhibition of cell migration was confirmed by the wound healing assay; PIC treatments impaired MDA-MB231 cell movement in a concentration-dependent manner (Fig. 1D). The inhibition of cell motility by PIC was not due to a decrease in cell viability because PIC up to 10 ng/ml did not alter cell viability within 36 hours of exposure (Fig. 1E). PIC-induced activation of PKR was verified by an increase in the phosphorylation of PKR (Thr446) and eIF2α (Ser51), a substrate of PKR in MDA-MB231 cells (Figs. 1F and G).

To determine whether PIC inhibition of cell migration was indeed mediated by PKR, we used a PKR inhibitor (PKR-I) to block PKR activity. As shown in Fig. 2A, PKR-I inhibited PIC-mediated phosphorylation of PKR and eIF2α. PKR-I blocked PIC-induced phosphorylation of p38 MAPK and pMK2, indicating that p38 MAPK/pMK2 was downstream of PKR (Figs. 2A and B). More importantly, PKR-I alleviated PIC-induced inhibition of cell migration (Fig. 2B). PKR-I also reduced PIC-mediated formation of lamellipodia (data not shown). These data indicated that activation of PKR suppressed the motility of breast cancer cells.

### Activation of PKR Impairs Lamellipodia Formation

The formation of lamellipodia at the leading edge of cells is characteristic of cells in motion. Since PIC inhibited cell motility, we sought to test whether PIC affected the formation of lamellipodia. MDA-MB231 cells were pretreated with PIC for 12 hours, allowed to attach to fibronectin for 3 hours and examined under the microscope. In the control group, the majority of cells formed a protrusive lamella (lamellipodia) (Figs. 3A and B); however, in the PIC-treated group, the number of cells with lamellipodia was significantly reduced. Since dynamic polymerization/depolymerization of the actin cytoskeleton is required to generate the force to form lamellipodia, we sought to determine whether PIC altered the organization of the actin cytoskeleton. In the control group, the actin cytoskeleton was cumulated at the lamellipodia and formed a polarized actin-rich leading edge (Fig. 3C). In the PIC-treated group, few cells displayed this feature. In addition, PIC also decreased cell spreading areas (Fig. 3D). Thus, PIC inhibited the reorganization of the actin cytoskeleton and impaired lamellipodia formation; these alterations may underlie PIC-induced inhibition of cell motility.

### Activation of PKR Inhibits Cofilin through p38 MAPK/MK2/LIMK Pathways

We next sought to determine the mechanisms for PIC-induced actin cytoskeleton reorganization and decreases in lamellipodia formation. Cofilin is an F-actin severing protein and plays an important role in the reorganization of the actin cytoskeleton. Cofilin activity is negatively regulated by phosphorylation at serine 3; phosphorylation of cofilin renders it unable to depolymerize F-actin, thereby stabilizing the cytoskeleton [22]. We examined the effect of PIC on cofilin activity. PIC increased the phosphorylation of cofilin (Ser3) (Figs. 4A and B). PIC-induced cofilin phosphorylation was confirmed by immunofluorescent staining (Fig. 5C). In the control group, active cofilin (non-phosphorylated form) was distributed at the leading edges of cells, i.e. lamellipodia (Fig. 4C). In the PIC-treated group, however, no active cofilin was observed at the leading edges. Since the localization of active cofilin at the leading edge is required for the initiation of cell movement [22], PIC-induced inhibition of cofilin activity and redistribution of cofilin may account for decreased lamellipodia formation and cell movement.

We next examined the potential signal pathways that regulate cofilin activity. LIM kinase 1 (LIMK1) is an important upstream kinase of cofilin that regulates the phosphorylation of cofilin [23]. LIMK activity is subjected to the regulation of p38 MAPK/MK2 pathways [24]. As shown in Fig. 4A, PIC induced phosphorylation of PKR (Thr446), LIMK (Thr505/508), p38 MAPK (Thr180/183), MK2 (Thr334) and cofilin (Ser3). PKR inhibitor blocked PIC-induced phosphorylation of p38 MAPK and MK2 (Figs. 2A and B), but the p38 MAPK inhibitor (SB203580) did not affect PKR phosphorylation (Figs. 5A and B), suggesting that PKR is upstream of p38 MAPK and MK2. Next, we sought to determine whether PIC-induced cofilin phosphorylation was mediated by p38 MAPK. As shown in Figs. 5A and B, SB203580 diminished PIC-induced phosphorylation of p38 MAPK, MK2, LIMK as well as cofilin. The finding was confirmed by immunofluorescent staining (Fig. 5C). These results suggested that p38 MAPK was upstream of LIMK and cofilin; PIC-mediated activation of p38 MAPK resulted in phosphorylation of LIMK and cofilin. Furthermore, SB203580 alleviated PIC-induced inhibition of cell migration (Fig. 5D). The involvement of PKR in p38/MK2 signaling was further supported by the study using PKR siRNA. As shown in Fig. 6, treatment of PKR siRNA blocked PIC-induced activation of p38/MK2. Taken together, these results suggested the activation of PKR suppressed cofilin activity through activation of the p38/MK2/LIMK pathway.

### Activation of PKR Inhibits Cell Membrane Ruffling

Cell membrane ruffling is a dynamic movement with rapid irregular vacillation of protrusion and withdrawal of a cell surface membrane. Cell membrane ruffling i.e. dynamic protrusion usually appears in the leading edge of a motile cell and is considered a dynamic parameter of cell motility [25]. An increase in membrane ruffling is positively associated with cell movement potential. To gain further insight into the relationship between PKR activation and cell motility, we investigated the effect of PIC on membrane ruffling using a time-lapse monitoring system. The cell membrane ruffling was recorded for 10 min in 10 second intervals. The kinetics of cell membrane ruffling was analyzed using kymography as previously described [14]. As shown in Fig. 7, PIC significantly decreased the rate of cell membrane ruffling. Both PKR inhibitor and SB203580 eliminated PIC-induced inhibition of membrane ruffling. These results further supported the findings presented above and indicated the PKR/p38 MAPK pathway regulated the motility of breast cancer cells.

## Discussion

PKR has been implicated in anti-tumor action due to its anti-proliferative and pro-apoptotic potential. Here, for the first time, we demonstrate that activation of PKR inhibits the motility of breast cancer cells. PKR activation suppresses lamellipodia formation, cell spreading and membrane ruffling. We further established the p38 MAPK/MK2/LIMK/cofilin signaling pathway is involved in PKR modulation of cell migration (Fig. 8).

### PKR and Cancer Cell Motility

PKR, the prototype of the eIF2α kinases, was first identified as an anti-viral serine/threonine kinase. Activation of PKR phosphorylates eIF2α at Ser51, resulting in inhibition of protein synthesis. Apart from inhibition of protein synthesis, PKR activation can favor either the induction of cell cycle arrest or apoptosis [10]. Therefore, there is an increasing interest to explore PKR’s anti-tumor potential [3], [7], [10], [26], [27]. Available evidence supports the notion that activation of PKR can inhibit tumour cell growth and may have therapeutic benefits. It has been proposed to develop strategies of selective PKR activation in cancer cells to kill tumor cells [26]. PKR is over-expressed in some human cancers, such as breast cancer, melanoma cells and colon cancer [10]. Although the implication of high levels of PKR in these cancer cells is unclear, the high expression of PKR may offer an opportunity to target PKR and optimize cancer cell toxicity. Our study demonstrates that PKR activation inhibits the migration of breast cancer cells independent of cell cycle arrest and the induction of apoptosis, suggesting PKR activity may negatively regulate cell mobility. This finding is consistent with the report showing there is an inverse relationship between the aggressiveness and the expression/activity of PKR in breast cancer cells [28], [29]. The current study focuses on breast cancer cells and shows that PKR activation impairs the migration of three aggressive breast cancer cell lines (BT474, MDA-MB231 and SKBR3). It remains to be determined whether the effect is general to all cell types. Cancer metastasis consists of multiple processes, and enhanced cancer cell motility is the hallmark of invasion and a critical step in each metastatic process [30]. Regardless whether PKR’s effect is cell type-specific or not, the *novel* finding provides a potential approach to developing strategies of cancer therapy targeting the PKR pathway, especially for those cancers overexpressing PKR.

### Signal Pathways that Mediate PKR Inhibition on Cell Motility

Cell motility initiates with cell protrusion, i.e. lamellipodia formation which directs cell migration and is controlled by actin remodelling. We demonstrate that PKR activation inhibits lamellipodia formation, cell spreading and membrane ruffling, indicating a disruption of actin cytoskeleton reorganization is a mechanism of PKR activation-induced inhibition on cell motility. The cooperation between cofilin and Arp2/3 is essential for this initial step [31]. Cofilin severs actin filaments to create free barbed ends from which new filaments are elongated by Arp2/3. The force of actin depolymerization/polymerization on the leading edge drives cell protrusion. The distribution of active cofilin within leading edges of cells is a prerequisite for actin depolymerization/polymerization which leads to cell migration. Alterations in overall activity of cofilin have been implicated in cancer metastasis and is directly associated with invasion and metastasis of mammary tumors [16], [17]. The activity of cofilin is regulated by phosphorylation; phosphorylation of cofilin at Ser3 inactivates cofilin and abolishes its actin-severing ability, therefore inhibiting the formation of lamellipodia [23], [32]. We demonstrate that PKR activation stimulates cofilin phosphorylation at Ser9, suggesting PKR activation impairs cell motility by inactivating cofilin (Fig. 8).

Phosphorylation of cofilin at Ser3 is regulated by LIM kinase (LIMK) and testicular protein kinase [23], [32]. Cofilin activity can also be regulated by dephosphorylation which is mediated by slingshot, chronophin and other phosphatases [17]. LIMK1 is activated through phosphorylation at Thr508 by downstream kinases of the Rho family GTPases, such as PAK, MRCK and ROCK [23], [33]–[36]. Alternatively, LIMK1 can be activated by MAPK-activated protein kinase 2 (MK2), a substrate of p38 MAPK [24]. Overexpression of active LIMK1 inhibits the motility of mammary cancer cells [37]. We demonstrate that PKR activation causes the phosphorylation of p38 MAPK, MK2 and LIMK1 (Thr508), suggesting that this pathway is involved in PKR regulation of cofilin activity.

PKR can activate p38 MAPK through interactions with mitogen-activated protein kinase kinase 6 (MKK6) [38]–[41]. Activation of p38 MAPK pathways is implicated in PKR activation-related immune response, apoptosis and cell cycle arrest [40]–[42]. We confirm that PKR activation results in the activation of p38 MAPK in MDA-MB231 cells because PKR inhibitor blocks PIC-mediated p38 MAPK phosphorylation, but a p38 MAPK inhibitor (SB203580) fails to modulate PKR activity. Although SB203580 does not affect PKR, it inhibits PIC-induced phosphorylation of MK2, LIMK1 and cofilin. PIC-induced phosphorylation of MK2, LIMK1 and cofilin is not affected by inhibitors for other MAPKs, such as ERKs and JNKs (data not shown). Taken together, these results indicate the PKR/p38 MAPK/MK2/LIMK1/cofilin pathway is responsible for PIC-mediated inhibition of cell motility. It is unclear whether p38 MAPK/MK2 regulates LIMK1 directly or indirectly. MK2 may directly interact and phosphorylate/activate LIMK1 [24]. However, it is reported that p38 MAPK activates RhoA/ROCK which are upstream of LIMKs by interacting with heat shock protein 27 (HSP27) in MDA-MB435 cells [43]. Kobayashi et al. confirm that p38 MAPK/MK2 activates HSP27 [24]. Therefore, it is also likely that p38 MAPK/MK2 indirectly regulates LIMK1 through the activation HSP27 and ROCK.

In summary, we demonstrate that PKR activation inhibits the migration of breast cancer cells and establishes an underlying signal transduction pathway that is responsible for PKR’s action. This finding supports the notion that targeting PKR is an attractive strategy for cancer therapy, especially for cancer cells overexpressing PKR, such as breast cancer, melanoma and colon cancer.
